# Chronic Flail Tricuspid Valve Related to Blunt Chest Trauma: A Case Report

**DOI:** 10.7759/cureus.26949

**Published:** 2022-07-17

**Authors:** Kevin Pink, Yongxia Qu

**Affiliations:** 1 Internal Medicine, New York-Presbyterian Brooklyn Methodist Hospital, Brooklyn, USA; 2 Cardiology, New York-Presbyterian Brooklyn Methodist Hospital, Brooklyn, USA

**Keywords:** valve regurgitation, transoesophageal echo, tricuspid valve regurgitation, blunt chest trauma, flail tricuspid valve

## Abstract

Tricuspid valve regurgitation (TR) is common in the adult population. A small part of this population is devoted to those who have a flail tricuspid leaflet. Flail TR can be caused by blunt chest trauma. Onset is often acute and severe and rarely seen in the form of isolated flail TR. Here, we present a rare encounter with chronic isolated tricuspid valve flail and severe TR related to blunt chest trauma.

## Introduction

Some degree of tricuspid valve regurgitation (TR) is present in 65-85% of adults with a prevalence of 1,600,000 patients with moderate to severe TR in the United States [1-2]. Typically, we divide TR into the categories of primary and secondary. Secondary or functional TR is caused by right ventricular dilation leading to leaflet tethering and tricuspid annulus dilation [2]. Secondary TR is more common accounting for approximately 90% of cases, whereas primary TR accounts for 10% [2]. A small part of this population is devoted to flail tricuspid valves [3]. Flail tricuspid leaflet refers to a prolapsed leaflet of the tricuspid valve with an excursion of the leaflet or chords in the right atrium during diastole [3]. Flail TR is most commonly caused by blunt chest trauma or iatrogenic chordal severing during right heart biopsy [3]. Non-traumatic causes include myxomatous, infective endocarditis, and congenital [3]. Common presenting signs and symptoms of TR can be nonspecific such as shortness of breath and fatigue. Onset is often acute and severe and rarely seen in the form of isolated flail TR [3]. Here, we present a rare encounter with chronic tricuspid valve flail and severe TR related to blunt chest trauma. The purpose of this report is to discuss differences in the chronicity of tricuspid flail, as well as, diagnosis and management. 

## Case presentation

**Figure 1:**
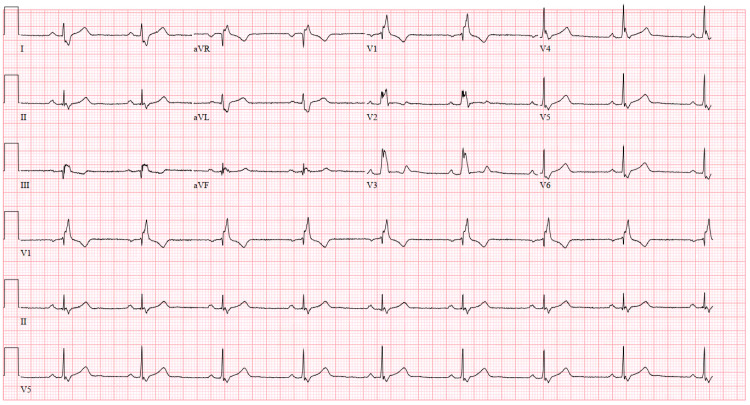
12 lead EKG: normal sinus rhythm, with right bundle branch block

A 43-year-old male with a past medical history of diet-controlled hyperlipidemia was referred after pre-op risk assessment for an outpatient endoscopic procedure for an abnormal EKG. The patient complained of dyspnea associated with palpitation when climbing four to five flights of stairs. He denied any other symptoms on the review of systems. He denied prior medical or surgical history. He was not on any medications and denied smoking, drinking, and recreational drug use. Family history was significant for atrial fibrillation and coronary artery disease. Laboratory studies were within normal limits. An electrocardiogram showed normal sinus rhythm with a right bundle branch block (Figure 1). 

**Figure 2:**
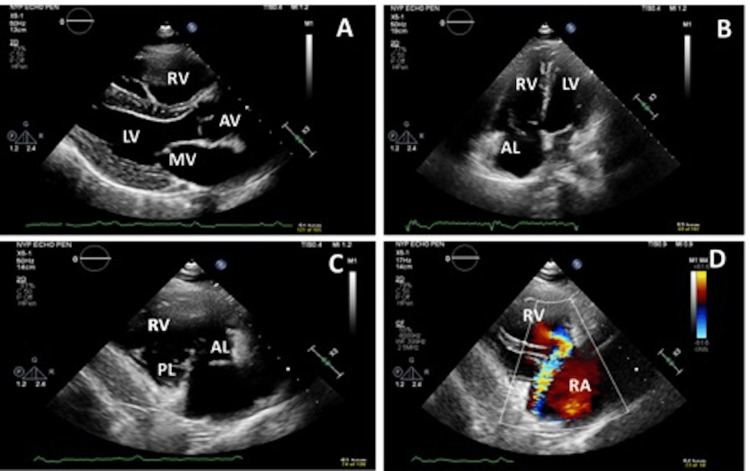
Transthoracic echocardiogram images. A. Parasternal long axis view showing normal mitral valve (MV), aortic valve (AV), and dilated right ventricle (RV); B. Four-chamber view showing dilated right ventricle and prolapse/flail of anterior leaflet of tricuspid valve; C. Right ventricle inflow view showing prolapse/flail of anterior leaflet of tricuspid valve; D. Severe eccentric tricuspid regurgitation

**Figure 3:**
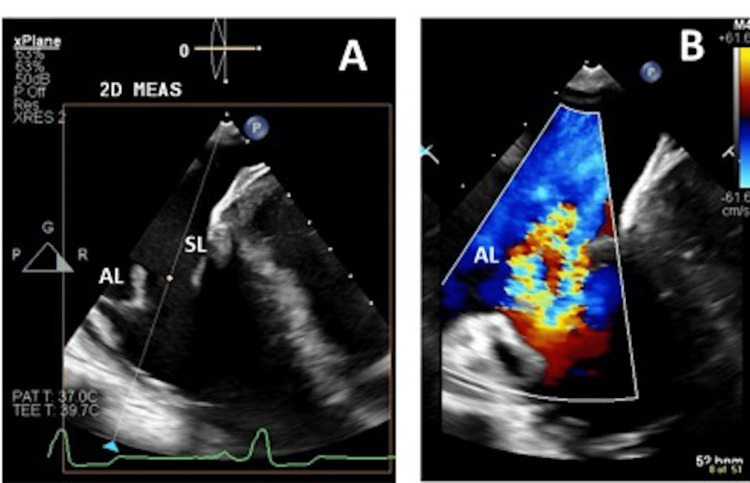
Still-frame images of transesophageal echocardiogram of the tricuspid valve regurgitation. A. Four-chamber view showing the flail of anterior leaflets of tricuspid valve; B. Color comparison images demonstrating the severe tricuspid regurgitation through the coaptation gap AL: anterior leaflet; SL: septal leaflet

Physical exam noted a holosystolic murmur, best heard at the left sternal border, radiating to the right lower sternal border. The intensity increased with inspiration. Large jugular V waves were seen during systole and peripheral pitting edema was absent. A transthoracic echocardiogram noted normal left ventricle size and function. The right ventricle was moderately dilated (Figures 2A, 2B) and systolic function was mildly reduced with a dilated right atrium. Severe eccentric tricuspid regurgitation secondary to tricuspid valve prolapse/flail was noted (Figures 2C, 2D). Pulmonary artery (PA) pressure was mildly elevated at 32 mmHg. Estimated PA systolic pressure is likely underestimated in the presence of severe tricuspid regurgitation secondary to rapid pressure equalization between the right ventricle and right atrium. Subsequent transesophageal echocardiogram (Figure 3) showed tricuspid anterior leaflet flail (3A) and severe tricuspid regurgitation (3B). The mitral valve, aortic valve, and pulmonic valve all appeared normal structure with no significant regurgitation.

Further history-taking revealed that while in his home country, 10 years prior, the patient was involved in a motorcycle collision with blunt chest trauma and was hospitalized in a remote, small local hospital. The patient was in a coma for a few days. The patient did not recall an echocardiogram being done at this time and was not aware of any abnormality with his heart. 

The patient subsequently underwent left and right heart catheterization with a tentative plan for surgical tricuspid valve repair vs percutaneous intervention. Left heart catheterization showed normal coronary anatomy. The right heart catheterization revealed mildly elevated right atrial pressure (7 mmHg), mildly elevated right ventricular pressures (28/12 mmHg), mildly elevated PA pressures (23/16), and a normal pulmonary capillary wedge pressure (11 mmHg). Cardiac output was 3.68 L/min and the cardiac index was 2.02 (L/min)/body surface area (BSA). 

## Discussion

Although the majority of adults have some degree of tricuspid valve regurgitation, severe TR as a complication flail leaflet is rare. Trauma is a large contributor to the causality of flail TR making up approximately 30% of all cases; however, the onset is often severe and acute. The pathophysiologic mechanism is believed to be a rapid deceleration force leading to increased right-sided cardiac pressures [4-5]. Cardiac injury can sometimes be overlooked after trauma due to provider propensity to focus on more overt injuries [4,6]. Given the fact the patient was not evaluated with echocardiography at the time of trauma, this is likely the case here. What is astonishing about this patient’s presentation is the body’s ability to compensate for severe TR over a period of 10 years. 

The initial evaluation of chest wall trauma based on Advance Trauma Life Support guidelines typically involves a focused assessment with sonography in trauma (FAST) exam [7]. This ensures some degree of cardiac imaging is obtained at the time of initial assessment so pathologies such as valvular regurgitation or cardiac tamponade are not missed [7]. It should be noted it takes a very experienced provider to identify tricuspid regurgitation with a point of care ultrasound. Tricuspid valve regurgitation is best evaluated by formal echocardiography as it allows us to better assess the severity and cause of TR. TR is typically graded based on valve hemodynamics and is divided by the American Heart Association into progressive TR (grade B), asymptomatic severe TR (grade C), and symptomatic severe TR (grade D). Progressive TR is defined by central jet <50% of the right atrium, vena contracta width <0.7 cm, effective regurgitant orifice <0.40 cm^2^, and regurgitation volume <45 mL [8]. Severe TR (grade C and D) is defined with a central jet > 50%, vena contracta width >0.7cm, effective regurgitant orifice >0.40 cm^2^, regurgitant volume >45 mL, a dense continuous wave signal with a triangular shape, and hepatic vein systolic flow reversal [8]. In severe TR, we also see a dilated right ventricle, a dilated right atrium, and C-v wave indicative of a systolic positive wave [8]. Both grades C and D will present with a clinical sign of elevated venous pressure; however, grade D will present with symptoms of dyspnea, ascites, fatigue and edema [8]. The patient in the case noted a vena contracta >1.0 cm, with symptom of dyspnea on exertion and, thus, was in grade D TR.

General medical management of severe tricuspid regurgitation involves treating right-sided heart failure with loop diuretics, pulmonary hypertension with vasodilators, and treatment of atrial fibrillation with rate and rhythm control [8]. It is generally recommended that all patients with severe TR undergoing left-sided cardiac surgery should undergo TR repair/replacement; however, isolated tricuspid valve repair is not as well defined [8]. It is recommended that patients with severe primary TR undergo isolated tricuspid valve repair; however, the strength of recommendation varies dependent on society [8-9]. Given that he developed reduced right ventricle systolic function, he was referred to CT surgery for tricuspid valve repair. He, however, declined open heart surgery and is being considered for percutaneous options, Triscend or Clasp.

## Conclusions

Flail tricuspid valve related to trauma is typically acute and severe in onset. However, in this case, we described how patients can experience more indolent courses of trauma-related TR. It remains important to identify flail tricuspid valve early on as it can often leads to right-sided heart failure and arrhythmia. Patients experiencing blunt chest trauma should be evaluated with echocardiography to ensure this valvular pathology is not missed. With early identification, patients can be appropriately triaged and evaluated for medical management or surgical intervention to avoid complications.
